# Comparing Same Day Sputum Microscopy with Conventional Sputum Microscopy for the Diagnosis of Tuberculosis – Chhattisgarh, India

**DOI:** 10.1371/journal.pone.0074964

**Published:** 2013-09-23

**Authors:** Priyakanta Nayak, Ajay M. V. Kumar, Mareli Claassens, Donald A. Enarson, Srinath Satyanarayana, Debashish Kundu, Kshitij Khaparde, Tarun K. Agrawal, Shankar Dapkekar, Sachin Chandraker, Sreenivas Achuthan Nair

**Affiliations:** 1 Office of the World Health Organization (WHO) Representative in India, WHO Country Office, New Delhi, India; 2 International Union against Tuberculosis and Lung Diseases (The Union), South-East Asia Regional Office, New Delhi, India; 3 Desmond Tutu TB Centre, Department of Paediatrics and Child Health, Stellenbosch University, Cape Town, South Africa; 4 State Tuberculosis Office, Directorate of Health Services, Raipur, Chhattisgarh, India; 5 Intermediate Reference Laboratory, Raipur, Chhattisgarh, India; Hopital Bichat Claude Bernard, France

## Abstract

**Background:**

The World Health Organization (WHO) recommends same day sputum microscopy (spot-spot) in preference to conventional strategy (spot-morning) for the diagnosis of smear positive tuberculosis with the view that completing diagnosis on a single day may be more convenient to the patients and reduce pre-treatment losses to follow-up.

**Methods:**

We conducted a cross-sectional study in seven selected district level hospitals of Chhattisgarh State, India. During October 2012 – March 2013, two sputum specimens (spot-early morning) were collected from consecutively enrolled adult (≥18 years) presumptive TB patients as per current national guidelines. In addition, a second sample was collected (one hour after the collection of first spot sample) from the same patients. All the samples were examined by ziehl-Neelsen (ZN) microscopy. McNemar’s test was used to compare statistical differences in the proportion smear positive between the two approaches (spot-spot versus spot-morning).

**Results:**

Of 2551 presumptive TB patients, 69% were male. All patients provided the first spot specimen, 2361 (93%) provided the second spot specimen, and 2435 (96%) provided an early morning specimen. 72% of specimens were mucopurulent in conventional strategy as compared to 60% in same day strategy. The proportion of smear-positive patients diagnosed by same day microscopy was 14%, as compared to 17% by the conventional method (p<0.001). A total of 73 (16.9%) potential cases were missed by the same day method compared to only 2 (0.5%) by the conventional method.

**Conclusion:**

Same-day microscopy method missed 17% of smear-positive cases and contrary to prior perception, did not increase the proportion of suspects providing the second sample. These findings call for an urgent need to revisit the WHO recommendation of switching to same-day diagnosis over the current policy.

## Introduction

Despite recent advances in rapid diagnostics, smear microscopy remains the most widely used test in low-income high tuberculosis (TB) burden countries [1]. The sputum smear-positive case detection rates globally and in India have been stagnant at a level below the 70% target set by the World Health Organization (WHO) [2]. Hence, efforts to improve the performance of sputum smear microscopy are a high priority for global TB control [3]. One reason for poor case detection could be the suboptimal use of sputum smear microscopy which currently involves the examination of two specimens collected over two days. A substantial proportion of patients fail to complete the two-day evaluation, especially in medical college hospitals and district level health facilities, owing to costs and inconvenience involved in multiple visits. If this process could be front loaded with both samples collected on the same day, it may reduce the number of patient drop-outs during diagnostic evaluation and increase the number of patients diagnosed and initiated on treatment [4,5]

The WHO recommends that countries that have successfully implemented the policy for a two-specimen case-finding strategy should consider switching to same-day diagnosis, especially in settings where patients are likely to be lost during the diagnostic process [6]. While sputum smear microscopy has been criticized for its low sensitivity relative to culture, the recent WHO recommendation of shifting to a two-smear strategy and same-day microscopy are likely to reduce sensitivity even further. Hence, countries have to test this strategy in their settings before adopting it. Most of the studies conducted hitherto have been under trial conditions and there is a lack of evidence from routine programme settings [7–10] and limited, conflicting information from India. A study from South India showed that same day microscopy was as effective as the conventional strategy [11]; whereas another study from North India showed that same day microscopy missed a considerable number of smear positive TB patients [12]. Both the studies were conducted in tertiary care hospitals with a limited sample size and were not representative of routine programme conditions. Hence, we aimed to assess if same-day (spot-spot) microscopy is as effective as the conventional (spot-morning) strategy in detecting sputum smear positive TB under programmatic settings in Chhattisgarh state, India. The specific objectives were to compare between same-day (spot-spot) microscopy and the conventional (spot-morning) strategy, (i) the number (proportion) of presumptive TB patients (erstwhile referred to as ‘TB suspects’) who provided two sputum specimens, (ii) the quality of specimens, (iii) the number (proportion) of sputum smear positive TB cases diagnosed, and (iv) the incremental yield of the second specimen.

## Methods

### Ethics considerations

All participants provided a written informed consent to participate in the study. Ethics approval was obtained by the Ethics Advisory Group of the International Union Against Tuberculosis and Lung Disease (The Union), Paris, France. Administrative approval to conduct the study was obtained from the Directorate of Health Services in the state of Chhattisgarh.

### Study design

A cross-sectional study.

### Study setting

In the state of Chhattisgarh in central India (population 25 million), 80% of the population live in rural areas and 30% are considered ‘tribal’ (as notified by the Government of India). There are 27 districts in the state with 326 designated microscopy centers (DMC). At district level, the quality of the smear microscopy is maintained through external quality assessment (EQA) by senior tuberculosis laboratory supervisors (STLS) under the guidance of district tuberculosis officers (DTO). This is further monitored and evaluated by the state intermediate reference laboratory (IRL) and the national reference laboratory (NRL). The EQA consists of on-site evaluation, un-blinded rechecking and random blinded rechecking of the selected slides. The study was carried out at seven district level hospitals (Bastar, Bilaspur, Dantewada, Dhamtari, Kanker, Mahasamund, Rajnandgaon). These hospitals have busy outpatient settings with trained laboratory technicians performing sputum microscopy. Presumptive TB patients are expected to provide two sputum specimens (spot and early morning) collected over two consecutive days which are examined for acid fast bacilli (AFB) by using the Ziehl-Neelsen staining technique. A patient is diagnosed as smear positive if either of the specimens is AFB positive as per WHO guidelines. In Chhattisgarh, of the 118 483 presumptive TB patients examined in 2012, 13 461 (11%) were smear positive.

### Study population and study period

Adult presumptive TB patients (aged ≥18 years) who had diagnostic smear microscopy in the seven district hospitals from October 2012 to March 2013 constituted the study population. The hospitals were selected purposively based on the area of work of the principal investigator.

### Data collection and data validation

**Figure 1:**
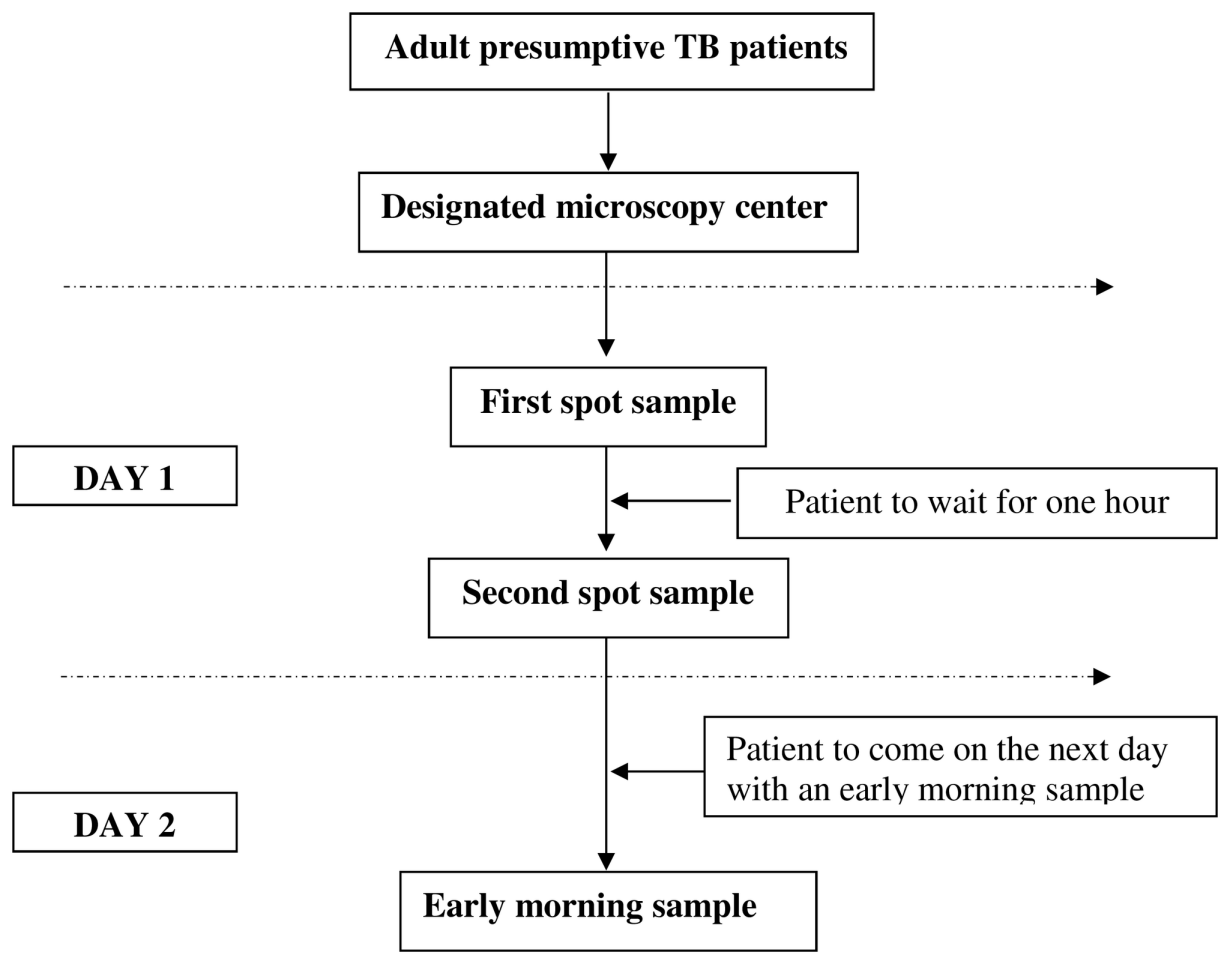
Data collection procedure during the study, Chhattisgarh, India, October 2012-March 2013.

The process is shown in Figure **1**. Consecutive presumptive TB patients attending DMCs were provided information on the study and requested to provide two ‘spot’ sputum samples one hour apart. The patient was requested to return the next day with an early morning sample. Specimens were assessed macroscopically for quality and smears were stained using the hot Ziehl-Neelsen (ZN) technique. Examination of the second spot sample was performed in a blinded manner by the LT. The senior tuberculosis laboratory supervisor (STLS), who was responsible for the blinding, labeled the second spot specimen with a different number before examination by the LT. The original number, new number and results were recorded in a study register. Smears were considered positive if it had >1 acid fast bacilli per 100 oil immersion fields examined. All smears were graded according to the WHO/International Union Against Tuberculosis and Lung Disease scale [13].

### Data variables

Information on the following variables – name of the hospital, age in completed years, sex, laboratory number, quality of specimen, sputum smear result – were extracted into a pre-tested structured data collection form by district level programme staff trained for the purpose. The laboratory supervisor was trained to review the data collection form for completeness and consistency which in turn was validated by the district TB officers (DTO) and medical consultants of the respective districts.

### Data entry and analysis

The data entry operators working for the national TB programme performed double data entry using EpiData entry software (Version 3.1, EpiData association, Odense, Denmark). The two databases were compared and validated for discrepancies, which were resolved through referral to the original data source. This finalized database was securely locked. A duplicate version of the finalized database, after removing personal identifiers, was used for statistical analysis using EpiData analysis software (Version 2.2.2.180). For the purposes of the analysis, sputum quality was graded in the following manner: (i) mucopurulent if any of two specimens was mucopurulent, (ii) bloodstained if one of the specimens was bloodstained and the other was salivary and (iii) salivary when both specimens were salivary. A patient was considered ‘smear positive’ if any of the two specimens was positive. To assess grading in case of two positive specimens, the higher grade was considered. Patients with missing smears were considered negative and included in analysis. We calculated incremental yield of the second specimen which referred to instances where the result of the first specimen was negative and second specimen was positive expressed as a percentage of all smear positives diagnosed. Here, the numerator was the number of patients with the serial results of two specimens being ‘NP’ (N-negative; P-positive) and the denominator was the number of all smear positive patients. We used the matched McNemar test for assessing statistical significance which was set at 5%.

## Results

Of 2551 presumptive TB patients enrolled, 1749 (69%) were male and the mean (SD) age was 45 (21) years.

Table **1** provides the comparison between the two approaches. All participants provided the first spot specimen, 2361 (93%) provided the second spot specimen, and 2435 (95%) provided an early morning specimen. 1833 (72%) of the participants provided mucopurulent specimens in conventional microscopy as compared to 1540 (60%) in same day microscopy. The proportion of smear positive patients diagnosed by same day microscopy was 360 (14%), as compared to 431 (17%) by the conventional method (p<0.001). The sputum positivity did not vary by centre.

**Table 1:** Comparison of conventional (spot-early morning) and same day sputum microscopy (spot-spot) in the diagnosis of tuberculosis, Chhattisgarh, India, October 2012-March 2013.

**Parameter**	**Conventional (spot-early morning**)	**Same Day (spot-spot**)	**P value**
**Total**	2551	2551	
**Number (%**)** who had both specimens examined**	2435 (96)	2361 (93)	<0.001
Quality of Sputum Specimen*			
Mucopurulent	1833 (72)	1540 (60)	<0.001
Blood Stained	29 (1)	30 (1)	
Salivary	685 (27)	975 (38)	
**Number (%**)** smear positive**	431 (17)	360 (14)	<0.001
Grading of positive smear**			
Scanty Positive	106 (25)	81 (23)	<0.001
1+ Positive	116 (27)	109 (30)	
2+ Positive	77 (18)	65 (18)	
3+ Positive	132 (31)	105 (29)	
Incremental yield of second specimen***	86 (20)	15 (4)	<0.001

* Quality was graded as mucopurulent if any of the specimens was mucopurulent and blood stained if one of the specimens was blood stained and other salivary. If both specimens were salivary, it was considered as salivary. Quality was not recorded for 6 patients under same day strategy and 4 patients for conventional strategy.

** This is among those with at least one positive smear. In case of two positive specimens, the higher grade was included

*** Incremental yield refers to instances where the result of the first specimen was negative and second specimen was positive expressed as a percentage of all smear positives diagnosed

The incremental yield of the second sputum specimen (instances when patients were negative on first specimen and positive only on second) was found to be 15 (4%) in same day microscopy as compared to 86 (20%) in conventional microscopy.

Considering the results of all three specimens, there were a total of 433 smear positive TB cases. Of this, 73 (17%) were missed by same day microscopy compared to 2 (1%) by the conventional method (Table **2**).

**Table 2:** Comparison of sputum smear microscopy results in conventional (spot-early morning) and same day sputum microscopy (spot-spot), Chhattisgarh, India, October 2012-March 2013 (N=2551).

**Strategy**	**Conventional (spot-early morning**)	
**Same Day (spot-spot**)	Smear Positive	Smear Negative
Smear Positive	358	2
Smear Negative	73	2118

## Discussion

This is the first study from India comparing same day microscopy with the conventional technique in routine program settings. Same day microscopy missed 17% of smear positive cases as compared to the conventional technique, thus the WHO recommendation of a same day sputum microscopy strategy may not be applicable in our setting.

The basis of the WHO recommendation comes from a meta-analysis and several studies which showed same day sputum microscopy has similar diagnostic accuracy as compared to the conventional technique [7–10]. While we are not sure of the reason for the difference in our findings, it might be related to the differences in patient characteristics and the spectrum of severity of TB in populations, which could be assessed indirectly using the distribution of smear quantification among smear positive TB patients [14]. In settings with a high TB burden, advanced disease and delayed access to care, most of the smear positive TB patients are likely to be ‘high positives’ (graded 2+ or 3+), and will be detected in a spot specimen. While early morning sputum specimens are known to more sensitive than spot specimens, the advantage is mitigated if most of the patients have advanced disease and are high positives [15].

Not all studies that have demonstrated equivalence between the two approaches have reported on the distribution of smear quantification [10]. In one study from Ethiopia which showed equivalence, none of the patients had scanty smears [16]. In contrast, nearly 50% of all smear positives in our study were low positives (either scanty or 1+) and hence the advantage of early morning specimen over second spot specimen is obvious. This may further indicate good access to services and patients reaching health facilities at an early stage in the state of Chhattisgarh. Introduction of the same day sputum microscopy may not be beneficial in such settings. Second, the higher yield of the early morning specimen may also be related to better quality of sputum specimens as compared to spot specimens. Third, most of the studies showing equivalent performance of same-day approach were done in well-controlled research settings with smear and culture examinations performed by well-trained personnel in reference laboratories [7–10]. In research settings, technicians are likely to spend more time reading the smears than in routine laboratories and hence are more likely to compensate for the difference between spot and overnight specimens; whereas, we conducted the study in routine settings and thus reflect operational realities.

The incremental yield of the second specimen was 4% in same day microscopy compared to 20% in the conventional method. Therefore, in situations where an early morning specimen cannot be obtained for any reason, a second spot will give at least some additional yield in sputum positivity and should be performed.

A surprising and counter-intuitive finding of our study was that the patient drop-out during diagnostic process was marginally higher with same-day approach as compared to conventional approach. The reason for this is not known and may require qualitative studies to assess why patients preferred to come on the morning of the second day to give their second sputum specimen instead of waiting for an hour on the same day. Inconvenience involved in waiting in over-crowded patient unfriendly health facilities and possible nosocomial exposure may have deterred the patients from waiting.

Since 2012, light emitting diode fluorescence microscopy (LED-FM) has been introduced in about 200 medical college microscopy centres in India. Given the better sensitivity (10% more) of LED-FM over ZN microscopy, it may be possible that LED-FM in spot specimens performs better in detecting acid-fast bacilli and the difference in performance between spot and early morning specimen may be lower [17]. Hence, it is possible that the same day approach could be as effective as conventional microscopy when LED-FM is used. This is a topic for future research.

There were a few limitations to our study. First, we conducted the study in seven purposively selected district hospitals and hence there may be an issue of generalizability of the findings. We deliberately chose busy district hospitals since we expected a higher chance of patient drop-out during the diagnostic process in these settings. If the same day approach did not confer any advantage in these settings, it is unlikely that it would be beneficial in more peripheral health institutions where most people are likely to provide two specimens. Second, all participants were provided detailed information including the need to provide second spot and early morning specimens during the process of informed consent. This might have influenced them to adhere to the diagnostic process and may not reflect the drop-outs that would have happened in routine conditions. Third, given the design of the study, we could not assess the effect of the different approaches on treatment uptake. Fourth, we did not have any information on HIV status of the participants or the proportion of TB patients with cavitary lesions and if that impacted the results. However, we feel that this might not have impacted greatly as the study was conducted in a low HIV prevalence setting (with an estimated HIV prevalence of 0.22% among adults aged 15-49 years in the year 2011) [18] and most of smear positive TB patients had their smears graded ‘low positive’ providing an indirect indication of likelihood of cavitary lesions.

## Conclusion

Same day microscopy missed 17% of smear-positive cases when compared to the conventional strategy. In contrast to prior studies, this strategy did not increase the proportion of suspects providing the second sample. The study findings call for a need to revisit the WHO recommendation of switching to same-day diagnosis. Countries should be cautious in their approach, pilot test the same day microscopy strategy and ascertain its usefulness before making decisions on a wider scale-up.

## References

[1] Partnership Stop TB (2006) The Global Plan to Stop TB, 2006-2015. Actions for life: towards a world free of tuberculosis. Int J Tuberc Lung Dis 10: 240-241. PubMed: 16562700. 16562700

[2] World Health Organization (2012) Global tuberculosis report 2012. World Health Organization Document WHO/HTM/TB/2012.6: 1-272.

[3] World Health Organization Document WHO/HTM/STB/2007.40: 1-100..

[4] Botha E, Den Boon S, Lawrence KA, Reuter H, Verver S et al. (2008) From suspect to patient: tuberculosis diagnosis and treatment initiation in health facilities in South Africa. Int J Tuberc Lung Dis 12: 936-941. PubMed: 18647454. 18647454

[5] Squire SB, Belaye AK, Kashoti A, Salaniponi FML, Mundy CJF et al. (2005) 'Lost' smear-positive pulmonary tuberculosis cases: where are they and why did we lose them? Int J Tuberc Lung Dis 9: 25-31. PubMed: 15675546. 15675546

[6] World Health Organization (2011) Same-day diagnosis of tuberculosis by microscopy. Policy statement. World Health Organization Document WHO/HTM/TB/2011.7: 1-11. 23586121

[7] Davis JL, Cattamanchi A, Cuevas LE, Hopewell PC, Steingart KR (2013) Diagnostic accuracy of same-day microscopy versus standard microscopy for pulmonary tuberculosis: a systematic review and meta-analysis. Lancet Infect Dis 13: 147-154. doi:10.1016/S1473-3099(12)70232-3. PubMed: 23099183. 3099(12)70232-3 . PII . doi:10.1016/S1473-3099(12)70232-3 23099183 10.1016/S1473-3099(12)70232-3 PMC3836432

[8] Hirao S, Yassin MA, Khamofu HG, Lawson L, Cambanis A et al. (2007) Same-day smears in the diagnosis of tuberculosis. Trop Med Int Health 12: 1459-1463. doi:10.1111/j.1365-3156.2007.01952.x. PubMed: 18076552. 18076552 10.1111/j.1365-3156.2007.01952.x

[9] Cuevas LE, Yassin MA, Al-Sonboli N, Lawson L, Arbide I et al. (2011) A multi-country non-inferiority cluster randomized trial of frontloaded smear microscopy for the diagnosis of pulmonary tuberculosis. PLOS Med 8(7): e1000443. doi:10.1371/journal.pmed.1000443. 21765808 10.1371/journal.pmed.1000443 PMC3134460

[10] Ramsay A, Yassin MA, Cambanis A, Hirao S, Almotawa A et al. (2009) Front-loading sputum microscopy services: an opportunity to optimise smear-based case detection of tuberculosis in high prevalence countries. J Trop Med, 2009 Article ID: 398767. doi:10.1155/2009/398767. PubMed: 20309419. 20309419 10.1155/2009/398767 PMC2836912

[11] Chandra TJ (2012) Same day sputum smear microscopy approach for the diagnosis of pulmonary tuberculosis in a microscopy centre at Rajahmundry. Indian J Tuberc 59: 141-144. PubMed: 23362710. 23362710

[12] Myneedu VP, Verma AK, Sharma PP, Behera D (2011) A pilot study of same data sputum smear examination, its feasibility and usefulness in diagnosis of pulmonary TB. Indian J Tuberc 58: 160-167. PubMed: 22533165. 22533165

[13] Aït-Khaled N, Alarcón E, Armengol R, Bissell K, Boillot F et al. (2010) Management of tuberculosis. A guide to the essentials of good practice. (sixth edition). 6 pp. 1-85.

[14] Hobby GL, Holman AP, Iseman MD, Jones JM (1973) Enumeration of tubercle bacilli in sputum of patients with pulmonary tuberculosis. Antimicrob Agents Chemother 4: 94-104. doi:10.1128/AAC.4.2.94. PubMed: 4208508. 4208508 10.1128/aac.4.2.94 PMC444512

[15] Andrews RH, Radhakrishna S (1959) A comparison of two methods of sputum collection in the diagnosis of pulmonary tuberculosis. Tubercle 40: 155-162. doi:10.1016/S0041-3879(59)80034-9. PubMed: 13793613. 13793613 10.1016/s0041-3879(59)80034-9

[16] Cambanis A, Yassin MA, Ramsay A, Squire SB, Arbide I et al. (2006) A one-day method for the diagnosis of pulmonary tuberculosis in rural Ethiopia. Int J Tuberc Lung Dis 10: 230-232. PubMed: 16499267. 16499267

[17] Cuevas LE, Al-Sonboli N, Lawson L, Yassin MA, Arbide I et al. (2011) LED fluorescence microscopy for the diagnosis of pulmonary tuberculosis: a multi-country cross-sectional evaluation. PLOS Med 8(7): e1001057. doi:10.1371/journal.pmed.1001057. PubMed: 21765809. 21765809 10.1371/journal.pmed.1001057 PMC3134458

[18] National AIDS Control Organization, National Institute of Medical Statistics (2013) Technical Report India HIV Estimates-2012. Directorate General of Health Services, Ministry of Health and Family Welfare, Government of India. Available: http://pib.nic.in/newsite/PrintRelease.aspx?relid=89785. Accessed: 2012 November 30.

